# Cancer treatment during the COVID-19 pandemic in the Kurdistan Region of northern Iraq

**DOI:** 10.3332/ecancer.2020.1096

**Published:** 2020-09-01

**Authors:** Sara Jamil Nidhamalddin, Rozhan Omer Hasan, Govar Othman Abubakr

**Affiliations:** 1 Medical Oncology Department, Hiwa Cancer Hospital, Sulaymaniyah, Iraq; 2 Clinical Hematology Department, Hiwa Cancer Hospital, Sulaymaniyah, Iraq

**Keywords:** COVID-19, neoplasms, communicable diseases, Iraq

## Abstract

The announcement of the COVID-19 pandemic by the World Health Organisation in March 2020 and the spread of coronavirus in most parts of the world, including Iraq, have posed severe challenges to the management of cancer patients both psychologically and logistically. Special experts at Hiwa Cancer Hospital, Iraq, took serious action aimed to decrease and prevent the spread of the virus among cancer patients while maintaining standard treatment protocols through compiling expert consensus, focusing on the prevention of COVID-19 and cancer patient management.

Following the worldwide spread of COVID-19, especially in neighbouring countries, experts at Hiwa Cancer Hospital in Iraq made some strategic decisions to decrease and prevent COVID-19 spread in cancer patients since this population group is prone to viral and bacterial infection more than the normal population due to immunocompromised status following active treatment and frequent visits to the hospital.

Hiwa Cancer Hospital is located in Sulaymaniyah city in the northeast of Iraq and southeast of Kurdistan. The city houses a population of 800,000 people as of 2016. Hiwa Cancer Hospital was built by the government sector and supported by both government and charitable donations. It is the only cancer hospital in Sulaymaniyah city. The hospital has 170 inpatient and 50 outpatient beds and yearly receives around 1,600–2,000 new adult haematology and oncology cases. About 100% of all patients are accepted for cancer management completely free of charge, irrespective of race or nationality.

At the beginning of the crisis, in late March 2020, there was a curfew all over the country. It was difficult for the patients to reach hospitals and seek medical care. The admission of new cancer patients was suspended along with prolonging neoadjuvant chemotherapy, stopping seeing follow-up cases and doing screening programs with delaying elective surgeries—this continued until late May. As time went by, we understood more that this problem is not a short duration crisis, and it is not logical to deprive patients from life-saving treatment. It was expected that there will be a gush of new cancer cases after the curfew is over, unfortunately, that is exactly what happened from late May when the government started to loosen the curfew and lockdown measures. As we all know, delaying diagnoses and treatment of new cancer cases will lead to their presentation at a more advanced stage which then leads to the reduced benefit of treatment and poor survival.

In order to continue cancer management according to the available facilities and based on the evidence available in the literature and guidelines [1–4], we followed the following polices from early June until the present time:

**(1) Decrease transmission of COVID-19**

The best way to continue standard medical and surgical care is through decreasing transmission of the virus and increasing protection through:

- Screening for COVID-19; anyone entering the hospital campus, whether they are a staff member, patient, patient’s companion or patient admitted in the ward, has to undergo initial screening by well-educated staff to triage suspicious cases.
  The first part of screening includes searching for symptoms associated with COVID-19 infection including fever, breathlessness and cough. The second part includes doing a SARS-cov2 PCR test for all those with:
  - Personal respiratory symptoms
  - One or more family member with respiratory symptoms in the past 14 days
  - History of residence or travel to a community with a high rate of COVID-19-positive cases in the past 14 days
  - History of exposure to a COVID-19-positive case in the past 14 days
  - In case of the positive result, the case will be isolated immediately and reported to a superior department in the hospital, and later on, the person will be referred to any COVID-19 dedicated hospital for further assessment and management.
- Personal protection: any person entering the hospital campus from the patient to their companion or hospital staff should be dealt with as an infected person to minimize the spread of the virus. Any person inside the hospital should wear surgical mask, gloves and surgical gown, keeping to required physical distancing and frequent hand washing. Special attention must be paid to high-risk patients (e.g., patients >65 years with severe comorbidities and those on active chemotherapy).
- Sanitise the hospital more frequently than routine hospital guidelines, and environmental ventilation, frequent disinfection of equipment and management of medical waste are measures believed to decrease nosocomial infection [1].
- Inpatient management: family visitors are strictly restricted, only one companion is allowed to stay with the patient and the patient and the companion must undergo daily screening. Meal delivery from outside the hospital is severely restricted, and the meals are prepared by specialized staff within the hospital and delivered by a nurse. When a patient is discharged, the room is sanitized and disinfected before admission of another patient
- Hospital staff training: arranging awareness training programs for all hospital staff to educate them on COVID-19 and its symptoms with preventive measures every other day with training on the appropriate use of PPE [6]

(b) Telephone call: to detect the positive cases of COVID-19 in the early stage, and the hospital set up a hot telephone line to:

- Register any cancer patient with COVID-19 symptoms, and in case of any suspicion of COVID-19, they will be contacted in order to get a sample from them. For positive RT-PCR test, all cancer treatments (chemotherapy, immunotherapy or targeted therapy) must be stopped temporarily, and the case has to be referred to specialized COVID-19 centres immediately. Although there is no accepted guideline for safe initiation or re-initiation of cancer treatment after COVID-19 infection, we started to re-challenge with anticancer therapy when the symptoms of COVID-19 have resolved for at least 48 hours, and the patient is at least 14 days from the onset of COVID-19 symptoms (or 14 days since specimen collection date if asymptomatic), or two negative PCR tests for COVID-19 collected at ≥24-hour interval [7which risk prediction tool should be used to guide admission disposition and management decisions? Key Messages from the Evidence Summary • Clinical risk prediction tools with acceptable performance have been identified for patients with various Non-COVID respiratory conditions who were admitted to general hospital wards (i.e., non-ICU and non-ED patients, 8]. Instructions must be given about not coming to the hospital unless in certain situations and the presence of specific indications to avoid unnecessary trips to the hospital.
- Careful scheduling for outpatients must be made by telephone call or any online measure before coming to the hospital to minimize the time in the waiting area
- Patient education: sending information about COVID-19, its signs and symptoms, ways of its transmission and protective measures through SMS and social media.

**(2) Maintaining cancer treatment routine**

The COVID-19 crisis is not a short-lived crisis, and we have to deal and cope with it. Treatment modification depending on international guidelines [2–5caused by a novel coronavirus (SARS-CoV-2] looks to be a better option rather than postponing treatment, which may harm the patient and be of higher economic burden on the hospital and government [6, 7]. We restarted to prescribe standard chemotherapy protocols with modifications after counselling and discussion with patients and their families, taking them on board in treatment decisions. There was a different route of administration whenever possible such as shifting to more oral agents, using less aggressive chemotherapy protocols and shorter treatment regimens and decreasing the frequency of immunotherapy regimens, for example, moving to 4-weekly or 6-weekly [2].

A special clinic was organized for phone consultation and psychological support. From this clinic, all patients were called and informed about this facility with an overwhelmingly positive response, providing all psychological support and answering any question or queries the patients have, and for them to feel safe while they are home, instructions were given about not coming to the hospital except in certain situations and specific indications to avoid unnecessary trips to the hospital, while palliative care and management of acute severe complications of cancer and relief of severe distress were maintained, providing adequate supply for pain management drugs at home [9].

The strategies and measures that we are following are local and are not unique and have many deficiencies. There are no courier services in the country, and the patients have to buy any necessary medication from outside the hospital; this was one of the main problems that we faced since, most of the times, the patient could not buy the necessary medications and had to visit the hospital due to economic issues. The country is an oil-dependent country, and an economic crisis was another negative impact of COVID-19. There were difficulties in retaining hospital employees and many lost their jobs, and the resultant limited number of healthcare staff disabled the facility of the administration of chemotherapeutic drugs at home and home care for patients who had advanced and end-stage disease.

## Conclusion

During the COVID-19 pandemic, we have to be flexible, patient and cautious, to share the experiences from different cancer centres around the world, to endorse best interim anticancer treatment regimens and be able to provide the best care for the patients until we reach a state that emergency measures put in place to address the COVID-19 pandemic are no longer necessary. It is highly recommended to have a virtual multidisciplinary team in place for decision-making, and each patient must be considered on an individual basis. In case of the modification of systemic anticancer treatment, using fewer immunosuppressive protocols, shifting from the intravenous route to oral or subcutaneous route to reduce visits and decreasing the frequency of immunotherapy protocols, it may be reasonable to postpone regular follow-up visits of patients not on active cancer treatment or to conduct those appointments via telemedicine.

## Conflicts of interest

The authors declare that no competing financial interests exist.

## Funding statement

This research was supported by Hiwa Cancer Hospital, Ministry of Health/Kurdistan Regional Government, Iraq.

## References

[1] ASCO (2020). For most updated content. https://www.asco.org/asco-coronavirus-information.

[2] NICE (2020). COVID-19 rapid guideline: delivery of systemic anticancer treatments. https://www.nice.org.uk/terms-and.

[3] Recomendation E ESMO recomendation. https://www.esmo.org/guidelines/cancer-patient-management-during-the-covid-19-pandemic?page=1.

[4] ESMO-Recommendations-Covid-19-General-Slide-Set.

[5] Lyu T, Song L, Jin L (2020). Expert consensus on the procedure of interventional diagnosis and treatment of cancer patients during the COVID-19 epidemic. J Interv Med.

[6] ILO (2020). With self-assessment checklist for RMG COVID-19 Management Guidance.

[7] Health A, Scientific SC, Group A (2020). COVID-19 scientific advisory group rapid response report.

[8] Curigliano G, Banerjee S, Cervantes A (2020). Managing cancer patients during the COVID-19 pandemic: an ESMO interdisciplinary expert consensus. Ann Oncol Off J Eur Soc Med Oncol.

[9] Zuo MZ, Huang YG, Ma WH (2020). Expert recommendations for tracheal intubation in critically ill patients with noval coronavirus disease 2019. Chinese Med Sci J = Chung-kuo i hsueh k’o hsueh tsa chih.

[10] Aghili M, Ghalehtaki R, Mousavi N (2020). Since January 2020 Elsevier has created a COVID-19 resource centre with free information in English and Mandarin on the novel coronavirus COVID-19. The COVID-19 resource centre is hosted on Elsevier Connect, the company’s public news and information.

[11] Ting FI, Benedict Sacdalan D, Sarita Abarquez H (2020). Treatment of cancer patients during the COVID-19 pandemic in the Philippines. Ecancermedicalscience.

[12] ESMO guidline Palliative care. https://www.esmo.org/guidelines/cancer-patient-management-during-the-covid-19-pandemic/palliative-care-in-the-covid-19-era.

